# Supplementary material for: Salinity Gradients Override Hydraulic Connectivity in Shaping Bacterial Community Assembly and Network Stability at a Coastal Aquifer–Reservoir Interface
Source: Microorganisms. 2025 Jul 8;13(7):1611. doi: 10.3390/microorganisms13071611 (PMC12300386; doi:10.3390/microorganisms13071611)
Supplement: Supplementary file 1 [file microorganisms-13-01611-s001.zip › microorganisms-3695232-supplementary.pdf]

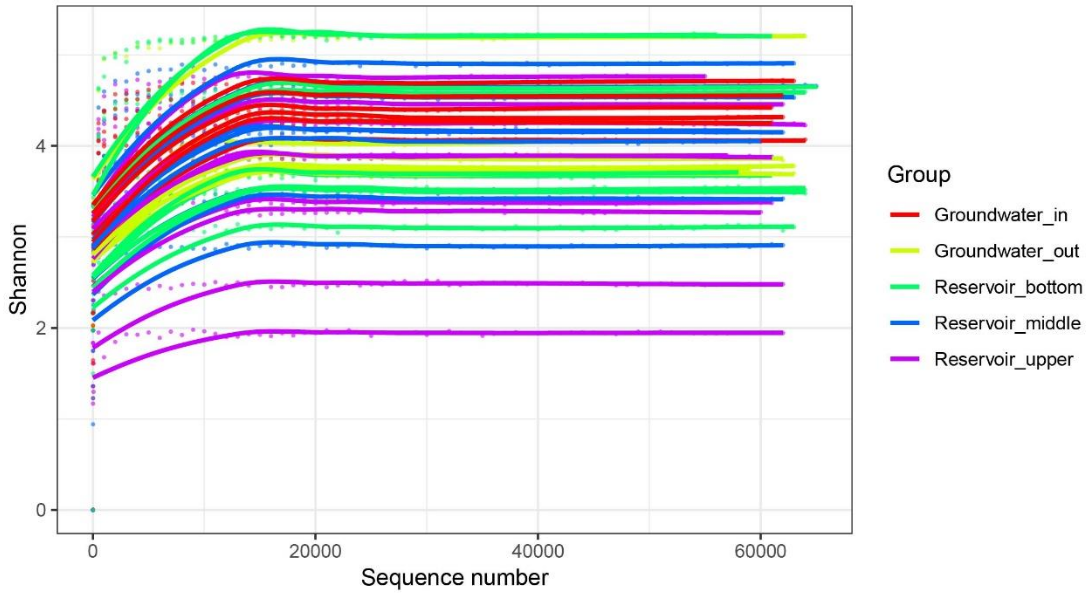

Figure S1. Rarefaction curve of bacterial community Shannon index reaches saturation stage with increasing sequencing depth.

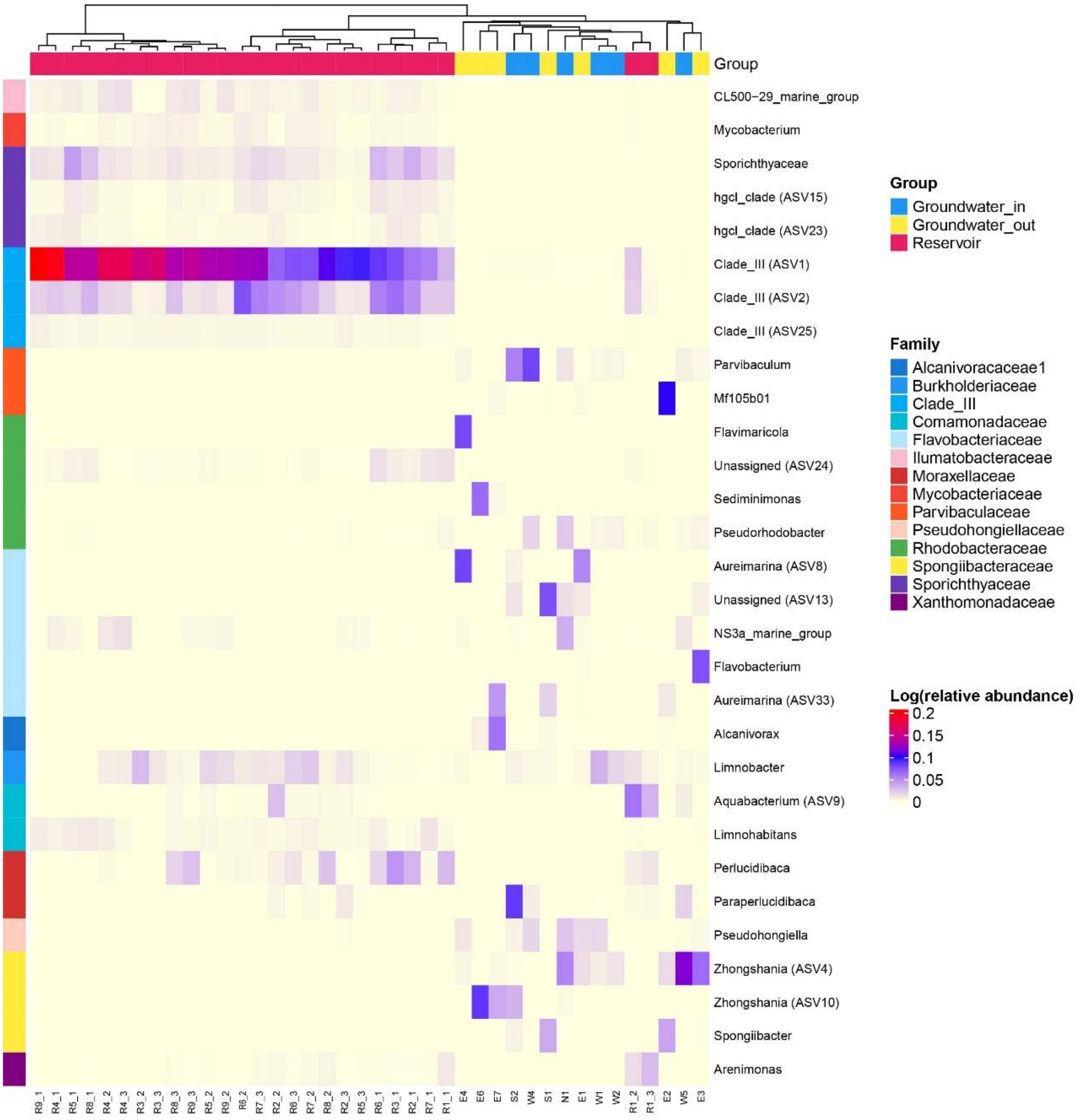

Figure S2. The heatmap shows the different abundance of the top 30 genera in the different sites of groundwater and layers of reservoir water. Suffixes of sampling sites in the reservoir represent water layers (_1: upper, _2: middle, _3: bottom).

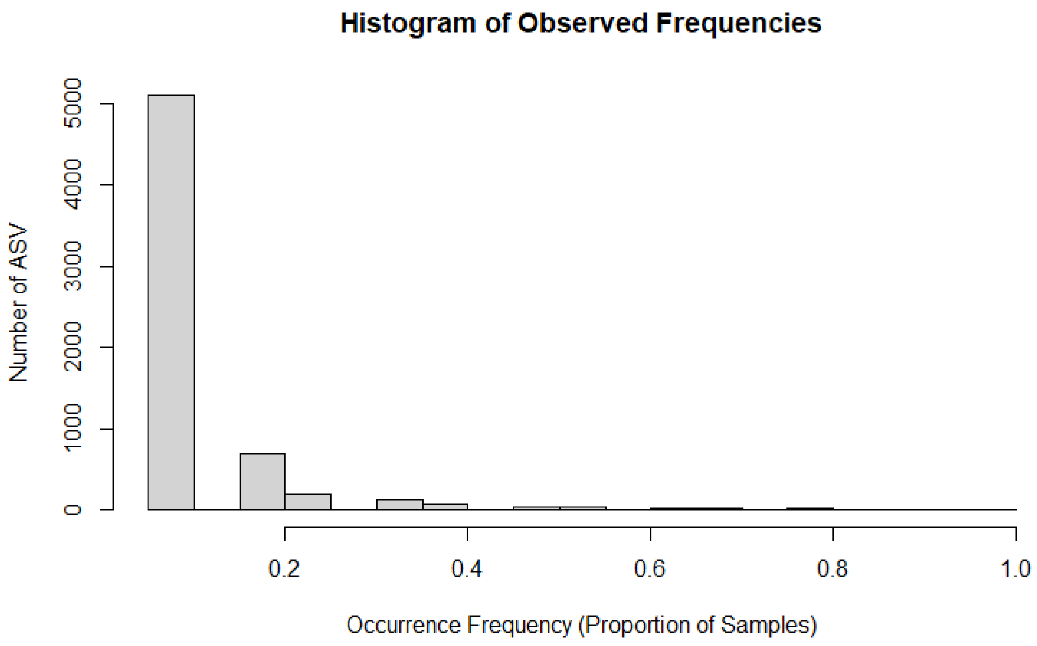

Figure S3. Distribution of ASV occurrence frequency in all groundwater samples.

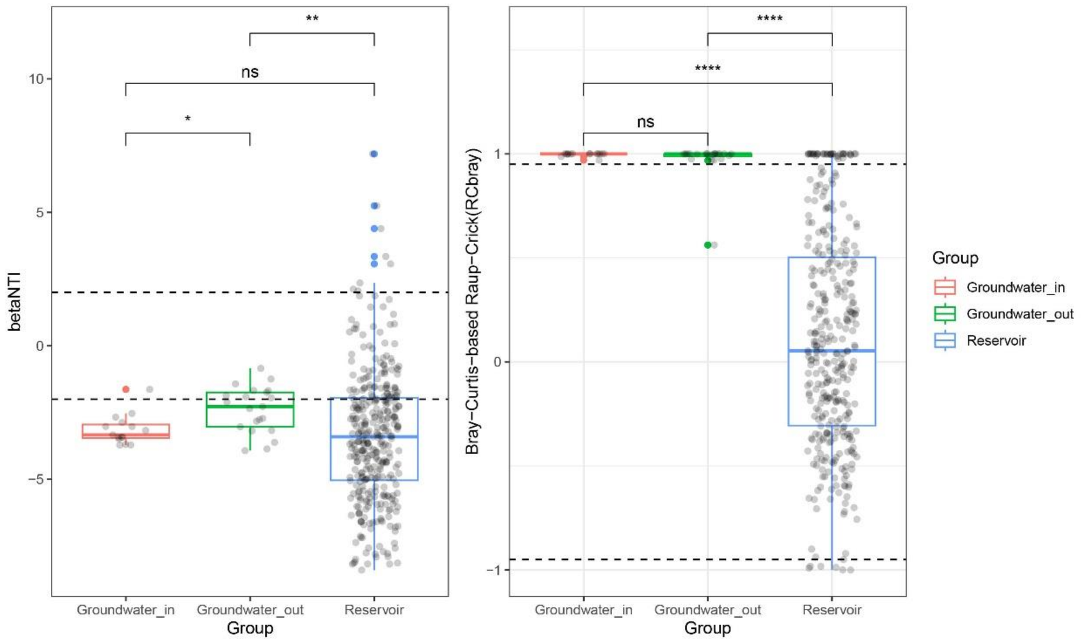

Figure S4. βNTI and Bray-Curtis-based Raup-Crick (RCbray) analyses of bacterial communities through null model among groundwater_in, groundwater_out and reservoir. Comparisons between abundant and rare taxa with the Wilcox test, (***p* < 0.01, ****p* < 0.001, *****p* < 0.0001). A βNTI>2 suggests that the heterogeneous selection of deterministic processes regulates the community assembly, while a βNTI<−2 suggests that the homogeneous selection of deterministic processes plays a dominant role in community assembly. A |βNTI| < 2 indicates that the observed discrepancies in phylogenetic diversity are primarily influenced by stochastic processes. These processes could be further classified into three types according to the RCbray metric: (1) when |βNTI| < 2 and RCbray < −0.95, this indicates homogenizing dispersal; (2) when |βNTI| < 2 and RCbray > 0.95, this reflects dispersal limitation; and (3) when |βNTI| < 2 and |RCbray| < 0.95, this denotes “undominated” processes, which are typically characterized by weak selection, weak dispersal, diversification, and/or genetic drift.

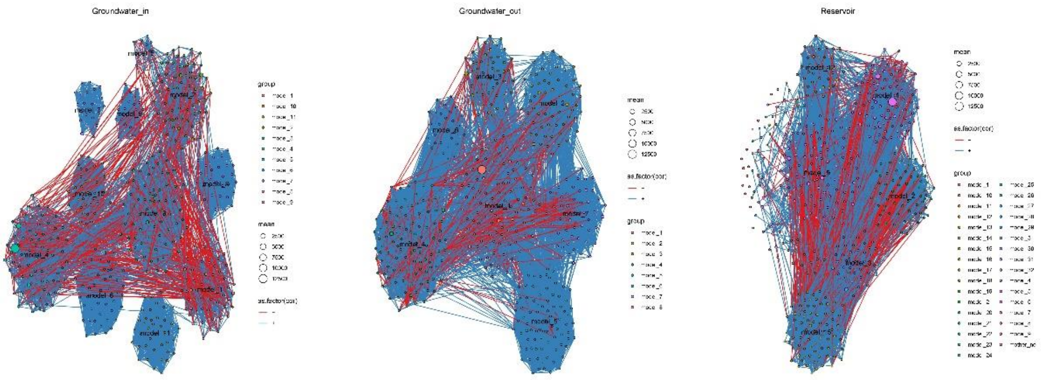

Figure S5. Co-occurring network of bacterial communities colored by different modules in the shallow groundwater (groundwater_in and groundwater_out) and reservoir. Each node represented an individual ASV, and the edges showed the correlation between two ASVs. The modules containing more than 5 ASVs in every network were displayed.

Table S1 Environmental hydrochemical variables in the groundwater_in, groundwater_out, three water layers (upper, middle, and bottom) of the reservoir, presented as mean ± standard deviation. TDS: total dissolved solids; TN: total nitrogen; TP: total phosphorus; DOC, dissolved organic carbon; COD: Chemical Oxygen Demand; Eh, oxidation-reduction potential.

Physicochemical factors	Groundwater_in (6)	Groundwater_out (7)	Reservoir		
			Bottom (9)	Middle (9)	Upper (9)
K^+ (mg/L)	41.52±39.94	192.01±136.45	16.83±8.22	13.70±5.31	11.91±3.96
Ca^{2+} (mg/L)	284.33±79.83	270.21±227.31	110.24±8.95	106.22±9.41	105.83±10.30
Na^+ (mg/L)	2493.33±825.26	7258.57±5310.52	702.33±439.65	534.22±267.86	441.33±204.01
Mg^{2+} mg/L)	418.67±150.47	966.86±772.04	124.26±54.11	101.93±34.39	90.43±29.43
Cl^- (mg/L)	3671.67±1098.95	12965.71±10392.27	1175.00±804.15	859.89±461.69	705.67±350.91
SO_4^{2-} (mg/L)	1785.83±1074.44	2125.57±1544.97	408.67±78.38	380.44±82.67	358.44±85.77
HCO_3^- (mg/L)	939.33±284.79	853.86±188.99	323.89±34.66	317.89±29.23	307.89±16.90
TDS (mg/L)	9830±2991	25223±18704	2861.22±1474.91	2314.30±926.30	2021.51±735.40
NH_4^+ mg/L)	0.89±1.22	0.98±0.79	0.32±0.39	0.23±0.12	0.25±0.12
TN (mg/L)	2.07±2.30	5.04±3.06	2.10±1.28	1.94±0.88	2.04±0.96
TP (mg/L)	0.07±0.05	0.18±0.17	0.08+0.05	0.06±0.02	0.05±0.01
DOC (mg/L)	49.33±5.15	58.76±23.90	49.67±3.92	51.05±4.48	6.82±1.64
COD (mg/L)	3.72±2.17	13.66±15.83	6.29±1.75	6.14±1.76	5.58±1.20
pH	7.48±0.30	7.46±0.14	7.87±0.23	7.96±0.21	7.89±0.18
Eh (mV)	184.45±5.68	190.56±4.02	138.51±5.75	137.41±5.34	139.40±4.76

Table S2 Hydrochemistry type in the shallow groundwater and reservoir water.

Sampling sites	Hydrochemical type		Sampling sites	Hydrochemical type
Groundwater_in			R7_1	SO_4-Cl-Na
N1	SO_4-Cl-Na		R8_1	SO_4-Cl-Na
W1	SO_4-Cl-Na		R9_1	Cl-Na
W2	Cl-Na-Mg		R1_2	SO_4-Cl-Na
W4	Cl-Na		R2_2	SO_4-Cl-Na
W5	Cl-Na		R3_2	Cl-Na
S2	Cl-Na		R4_2	Cl-Na
Groundwater_in			R5_2	Cl-Na
S1	Cl-Na		R6_2	SO_4-Cl-Na
E1	Cl-Na		R7_2	SO_4-Cl-Na
E2	Cl-Na		R8_2	Cl-Na
E3	Cl-Na		R9_2	Cl-Na
E4	Cl-Na		R1_3	SO_4-Cl-Na
E6	Cl-Na		R2_3	Cl-Na
E7	Cl-Na		R3_3	Cl-Na
Reservoir			R4_3	Cl-Na
R1_1	SO_4-Cl-Na		R5_3	Cl-Na
R2_1	SO_4-Cl-Na		R6_3	SO_4-Cl-Na
R3_1	SO_4-Cl-Na		R7_3	SO_4-Cl-Na
R4_1	Cl-Na		R8_3	Cl-Na
R5_1	Cl-Na		R9_3	Cl-Na
R6_1	SO_4-Cl-Na			

Table S3 Pairwise comparisons test by PERMANOVA (permutational multivariate analysis of variance) based on Bray-Curtis dissimilarity matrix in the Principal coordinates analysis (PCoA), between different water layers of reservoir, within the groundwater_in, groundwater_out, and water layers of reservoir using the "pairwiseAdonis" package in R.

Pairwise comparisons	R^2	*p*. value	*p*. adjusted
Reservoir_upper vs. Reservoir_middle	0.197	0.002	0.003
Reservoir_upper vs. Reservoir_bottom	0.184	0.002	0.003
Reservoir_middle vs. Reservoir_bottom	0.020	0.997	0.997
Groundwater_in vs. Groundwater_out	0.123	0.013	0.013
Groundwater_in vs. Reservoir	0.266	0.001	0.002
Groundwater_out vs. Reservoir	0.259	0.001	0.002
Groundwater_in vs. Reservoir_upper	0.411	0.001	0.002
Groundwater_in vs. Reservoir_middle	0.364	0.001	0.002
Groundwater_in vs. Reservoir_bottom	0.333	0.002	0.003
Groundwater_out vs. Reservoir_upper	0.363	0.001	0.002
Groundwater_out vs. Reservoir_middle	0.329	0.001	0.002
Groundwater_out vs. Reservoir_bottom	0.300	0.001	0.002

Table S4. Topological properties of the co-occurrence networks for bacterial communities in the groundwater (entire, groundwater_in, groundwater_out) and reservoir water.

**Properties parameters**	**Entire groundwater**	**Groundwater_in**	**Groundwater_out**	**Reservoir**
Nodes	500	500	500	483
Edges (links)	7519	7306	12182	7799
Positive edges	7032	5995	11142	6501
Negative edges	487	1311	1040	1298
Average degree	30.08	29.22	48.73	32.29
Average path length	1.78	2.29	2.19	1.71
Network diameter	3.79	4.22	4.74	4.66
Network density	0.06	0.06	0.10	0.07
Clustering coefficient	0.51	0.75	0.73	0.48
Centralization degree	0.06	0.04	0.08	0.16
Centralization betweenness	0.02	0.01	0.03	0.02
Centralization closeness	0.12	0.76	0.19	0.20
Network modularity	0.58	0.79	0.66	0.44
Modularity_random	0.11	0.12	0.08	0.11
RM (Relative modularity)	4.28	5.42	7.35	2.84

Table S5 Taxonomic information of keystone species from the network in the groundwater_out and reservoir water

ASV_ID	Node roles	Module_ID	Phylum	Class	Order	Family	Genus
**Groundwater_out**							
ASV76	Connectors	4	Proteobacteria	Gammaproteobacteria	Pseudomonadales	Moraxellaceae	
ASV344	Connectors	4	Bacteroidota	Bacteroidia	Flavobacteriales	Cryomorphaceae	uncultured
ASV467	Module hubs	4	Proteobacteria	Gammaproteobacteria	Pseudomonadales	Pseudomonadaceae	Pseudomonas
ASV715	Connectors	4	Proteobacteria	Gammaproteobacteria			
ASV1548	Module hubs	4	Dependentiae	Babeliae	Babeliales	Babeliaceae	Babeliaceae
ASV559	Module hubs	4	Proteobacteria	Alphaproteobacteria	Caulobacterales	Parvularculaceae	Hyphococcus
ASV284	Module hubs	4	Proteobacteria	Alphaproteobacteria	Caulobacterales	Hyphomonadaceae	Hyphomonas
ASV579	Connectors	5	Proteobacteria	Gammaproteobacteria	Alteromonadales	Pseudoalteromonadaceae	Pseudoalteromonas
ASV34	Connectors	5	Proteobacteria	Gammaproteobacteria	Burkholderiales	Burkholderiaceae	Polynucleobacter
ASV1122	Module hubs	6	Bacteroidota	Bacteroidia	Flavobacteriales	Flavobacteriaceae	Aureimarina
ASV1254	Module hubs	6	Proteobacteria	Alphaproteobacteria	Parvibaculales	Parvibaculaceae	Parvibaculum
ASV794	Connectors	3	Proteobacteria	Alphaproteobacteria	Rhodobacterales	Rhodobacteraceae	
ASV1727	Connectors	3	Proteobacteria	Alphaproteobacteria	Parvibaculales	Parvibaculaceae	Tepidicaulis
ASV59	Connectors	2	Bacteroidota	Bacteroidia	Flavobacteriales	Flavobacteriaceae	
ASV1413	Connectors	2	Proteobacteria	Gammaproteobacteria	Burkholderiales	Comamonadaceae	
ASV1649	Connectors	2	Proteobacteria	Alphaproteobacteria	Rhodobacterales	Rhodobacteraceae	Aestuariicoccus
**Reservoir**							
ASV80	Connectors	2	Proteobacteria	Gammaproteobacteria	Burkholderiales	MWH-UniP1_aquatic_group	MWH-UniP1_aquatic_group
ASV119	Connectors	2	Bacteroidota	Bacteroidia	Sphingobacteriales	NS11-12_marine_group	NS11-12_marine_group

ASV196	Connectors	2	Proteobacteria	Gammaproteobacteria	Burkholderiales	MWH-UniP1_aquatic_group	MWH-UniP1_aquatic_group
ASV84	Module hubs	2	Proteobacteria	Gammaproteobacteria	Burkholderiales	Comamonadaceae	Polaromonas
ASV117	Connectors	2	Proteobacteria	Gammaproteobacteria	Burkholderiales	Comamonadaceae	Limnohabitans
ASV788	Connectors	2	Bacteroidota	Bacteroidia	Flavobacteriales	Crocinitomicaceae	Fluviicola
ASV175	Connectors	2	Proteobacteria	Gammaproteobacteria	Burkholderiales	Comamonadaceae	Hydrogenophaga
ASV57	Module hubs	3	Actinobacteriota	Actinobacteria	Frankiales	Sporichthyaceae	hgcI_clade
ASV64	Module hubs	3	Actinobacteriota	Actinobacteria	Frankiales	Sporichthyaceae	Candidatus_Planktophila
ASV101	Connectors	3	Bacteroidota	Bacteroidia	Flavobacteriales	Crocinitomicaceae	Crocinitomix
ASV70	Connectors	4	Actinobacteriota	Actinobacteria	Micrococcales	Microbacteriaceae	ML602J-51
ASV776	Connectors	4	Bacteroidota	Bacteroidia	Chitinophagales	Saprospiraceae	uncultured
ASV106	Connectors	4	Proteobacteria	Gammaproteobacteria	Cellvibrionales	Halieaceae	
ASV156	Module hubs	4	Proteobacteria	Gammaproteobacteria	Cellvibrionales	Halieaceae	OM60(NOR5)_clade
ASV171	Connectors	4	Proteobacteria	Gammaproteobacteria	Burkholderiales	Comamonadaceae	Rhodoferax
ASV316	Module hubs	4	Actinobacteriota	Acidimicrobiia	Microtrichales	Ilumatobacteraceae	Ilumatobacter
ASV252	Connectors	4	Bacteroidota	Bacteroidia	Chitinophagales	Saprospiraceae	Lewinella
ASV212	Connectors	4	Proteobacteria	Alphaproteobacteria	Caulobacterales	Caulobacteraceae	Caulobacter
ASV721	Connectors	4	Proteobacteria	Alphaproteobacteria	Micavibrionales	uncultured	uncultured
ASV470	Connectors	4	Bacteroidota	Bacteroidia	Flavobacteriales	NS9_marine_group	NS9_marine_group
